# Higher Blood 25(OH)D Level May Reduce the Breast Cancer Risk: Evidence from a Chinese Population Based Case-Control Study and Meta-Analysis of the Observational Studies

**DOI:** 10.1371/journal.pone.0049312

**Published:** 2013-01-30

**Authors:** Peizhan Chen, Mian Li, Xiaoli Gu, Yanling Liu, Xiaoguang Li, Chenglin Li, Yuan Wang, Dong Xie, Fudi Wang, Chen Yu, Jingquan Li, Xinlei Chen, Ruiai Chu, Jianmin Zhu, Zhouluo Ou, Hui Wang

**Affiliations:** 1 Key Laboratory of Nutrition and Metabolism, Institute for Nutritional Sciences, Shanghai Institutes for Biological Sciences, Chinese Academy of Sciences, Graduate School of the Chinese Academy of Sciences, Shanghai, P. R. China; 2 Breast Cancer Institute, Cancer Hospital, Department of Oncology, Shanghai Medical College, Fudan University, Key Laboratory of Breast Cancer in Shanghai, Shanghai, P. R. China; 3 Shanghai Xuhui Central Hospital, Shanghai, P. R. China; MOE Key Laboratory of Environment and Health, School of Public Health, Tongji Medical College, Huazhong University of Science and Technology, China

## Abstract

Experimental data suggest a protective effect of vitamin D on breast cancer; however, epidemiologic results remain inclusive. With a Chinese population-based case-control study and meta-analysis of the observational studies, we here systematically evaluated the association of blood 25(OH)D level and breast cancer risk. With 593 breast cancer cases and 580 cancer-free controls from Shanghai, China, we found that 80% of the normal women had severe vitamin D deficiency (less than 20 ng/mL) and 15.2% had mild deficiency (20 to 30 ng/mL) and only 4.8% of women had sufficient vitamin D level (>30 ng/mL) while the proportion was 96.1%, 3.2% and 0.7% respectively for the breast cancer patients. Compared to those with the lowest quartile of plasma 25(OH)D level, women with highest quartile 25(OH)D level showed a significant decreased breast cancer risk (Q4 vs.Q1: OR = 0.10, 95% CI = 0.06–0.15) and every 1 ng/ml increment of plasma 25(OH)D level led to a 16% lower odds of breast cancer (OR = 0.84, 95% CI = 0.81–0.87; P<0.001). From the meta-analysis of the observational studies, we found that women with highest quantile of blood 25(OH)D level was associated with a significantly reduced breast cancer risk compared to those with lowest quantile of blood 25(OH)D level for the 11 nested case-control and retrospective studies (pooled OR = 0.86, 95% CI = 0.75–1.00) and 10 case-control studies (7 population based, OR = 0.35, 95% CI = 0.24–0.52; 3 hospital based, OR = 0.08, 95% CI = 0.02–0.33). These results suggest that vitamin D may have a chemo-preventive effect against breast cancer.

## Introduction

Vitamin D is a fat-soluble vitamin that exists in a few kinds of foods and is mainly synthesized by the skin subsequent to sun exposure from 7-dehydrocholesterol [1], [2]. After absorption or synthesis, vitamin D is converted into 25(OH)D in the liver, which is the major storage form of vitamin D. The plasma 25(OH)D level has been widely recognized as the indicator of vitamin D status for the human body [3]. In the kidney, 25(OH)D is 1α-hydroxylated by mitochondrial 1α-hydroxylase to yield 1α,25-(OH)_2_D, the active form for vitamin D [4]. In the cells, the molecule 1α,25-(OH)_2_D binds to the vitamin D receptor (VDR), a member of steroid hormone receptor family which exerts transcriptional activation or repression of target genes activity through interaction with other co-factors [5]. It has been well established that vitamin D is essential for Ca^2+^ and Pi transport and bone mineralization in the human body and vitamin D deficiency leads to osteoporosis, rickets, fracture and other bone diseases [6], [7]. Recently, epidemiological studies have suggested that vitamin D deficiency is a global epidemic which may increase the risk of certain types of chronic diseases such as metabolic syndrome [8], diabetes [9], cardiovascular diseases [10], mental diseases [11] and immune response dysfunctions [12].

Accumulating evidence suggest that vitamin D and its downstream signaling pathways are involved in the proliferation, differentiation, apoptosis, angiogenesis, and metastasis for the cancer cells [13]. Vitamin D deficiency may be associated with increased risk for colorectal [14], prostate [15], and lung cancer [16]. Studies also found that lower 25(OH)D level in the lung [17], breast [18], colorectal cancer [19] and non-Hodgkin’s lymphoma [20] patients could be an independent prognosis factor for poorer clinical outcomes. VDR is expressed both in normal and transformed mammary cells and the vitamin D signaling pathway has potential protective effects against breast cancer tumorigenesis. The VDR knockout mice showed an increased incidence of mammary gland hyperplasia and a higher percentage of hormone independent tumors with squamous differentiation compared to the wide type mice when induced with the dimethylbenzanthracence [21]. VDR ablation also enhanced the tumorigenesis for the MMTV-neu transgenic model of breast cancer with shortened latency, increased incidence of mammary tumor formation and worse prognosis [22]. Epidemiological studies have been conducted to assess whether high vitamin D intake is capable of reducing the incidence of breast cancer. Although the conclusions from individual studies are inconclusive, the pooled results from the meta-analysis study suggest that higher vitamin D intake may reduce the breast cancer risk [23]. As an indicator of vitamin D status of human body, many case-control studies and nested case-control studies have evaluated the relationship between the circulating of 25(OH)D level and breast cancer risk. To date, population and hospital based case-control studies have suggested an inverse relationship between 25(OH)D level and breast cancer risk; however, results from the prospective nested case-control studies have been inconclusive. For there is a lack of the data for the Chinese population and the conclusions from the observational studies were inconclusive, we conducted a population based case-control study in the Chinese population and also performed a systematic meta-analysis study to evaluate the association between blood 25(OH)D level and the breast cancer risk.

## Materials and Methods

### Study Populations

593 newly diagnosis breast cancer patients aged between 30 and 87 years old were recruited at the Zhongshan tumor hospital during March, 2006 to July, 2007 with complete medical records. The patients were genetically unrelated ethnic Han Chinese from Shanghai and the surrounding regions. All patients had been histopathologically diagnosed with primary breast cancer and not yet treated. A 5-ml blood sample was provided by the patients before any treatment after the written informed consent was obtained. Patients were excluded if they had received any treatment for the breast cancer, or were not Chinese Han population, or with any other cancer disease.

The 580 unrelated female control samples were randomly extracted from the women who have participated in the Breast Cancer Screening Project of the same hospital during the year 2005 to 2008 at Minhang district of Shanghai, and the controls reported no previous cancer history and were further confirmed with no evidence of breast cancer, or any suspicious precancerous lesions of the breast by the physicians. The controls were Chinese Han population and between 25 to 85 years old. A 5-ml of blood sample was also collected for the controls and the date was recorded. The controls were matched with the breast cases with ±5 years old and the time of blood collection (±3 months).

All the participants were personally interviewed with the pre-trained interviewers to complete the structured questionnaire that evaluated the potential breast cancer factors for women, including age, age at menarche, age at first birth, marital status, and the first-degree relatives’ history of breast cancer. For the menopausal status, women were classified as postmenopausal if they affirmed that their menstruations had ceased or had no menstrual history during the past 12 months or more. Those with normal menstrual cycle or still with menstrual history during the past 12 months were assessed as pre-menopausal/peri-menopausal status as defined [24].

### Ethics Statement

This project has been approved by the Scientific and Ethical Committee of the Cancer Hospital of Fudan University. All of the samples were collected with a written informed consent provided by the participants.

### 25(OH)D Level Determination

After collection, all the blood samples were centrifuged at 3000g for 15 minutes and the plasma were immediately storage at −20°C in aliquots until use. The plasma 25(OH)D level was determined using a radioimmunoassay kit (DiaSorin, Stillwater, MN) according to the standard procedure. All of the coefficients of variation for within-assay and between-assay were less than 10%.

### Study Selection for the Meta-analysis

We systematically searched the PubMed and MEDLINE databases for eligibility studies that have been published online up to September 2012. We used the term ‘‘breast cancer’’ in combination with ‘‘vitamin D,’’ or ‘‘25-hydroxyvitamin D” and “plasma” or “serum” to identify the studies that have evaluated the blood 25(OH)D level and breast cancer risk. References of the included studies and related reviews were checked to identify any missing data in the database search. Only studies reported in English were included in our meta-analysis.

Eligibility studies should have reported blood 25(OH)D level in quantiles and the corresponding risk estimates and their 95% confidential intervals (CI), or provided the estimates for the highest quantile in contrast with the lowest quantile of blood 25(OH)D, or provided sufficient data that could be used to calculate the risk estimate and its 95% CI between the quantiles. If overlapping populations were identified between the studies, only the most complete data were included in the meta-analysis. Descriptive characteristics about each study, when available, were extracted from the published reports: first author’s last name, the year of publication, country of the study performed, study design, the sample size, the age range of the subjects, blood 25(OH)D level, the risk estimates that reflected the greatest degree of control for potential confounders with their corresponding confidence intervals, and the adjusted covariates.

### Statistical Analysis

The χ^2^ test (for categorical variables) and Student’s t-test (for continuous variables) were used to evaluate the differences in demographic characteristics and selected variables. To assess the seasonal distribution of vitamin D level in the cases and controls, the sample collect date was categorized into spring (March to May), summer (June to August), autumn (September to November) and winter (January, February and December). Comparisons of the vitamin D level between cases and controls and in different seasons were done using the nonparametric Wilcoxon tests. To determine the association of vitamin D status and breast cancer risk, we firstly divided the 25(OH)D level into quartiles according to its distribution in the control group. The multivariable conditional logistic regression was used to calculated the odds ratios (ORs) and their 95% confidential interval (95% CI) for quartiles Q2 through Q4 compared with the lowest quartile Q1 with or without the adjustment of the co-variants of age, age at first birth, age at menarche, menopausal status, first-degree family history of breast cancer and use of oral contraception. We also assessed the 25(OH)D level as continuous variants (per 1 ng/ml increment) to evaluate its association with breast cancer risk.

For the meta-analysis, the risk estimate OR and its 95% CI was extracted or calculated from each study. To establish the appropriate weighting for each study, the SE for each logarithm OR was calculated and each study was weighted using the generic inverse variance approach. The DerSimonian and Laird random-effects method which considers the variability within and between studies was used to calculate the pooled estimate [25]. Statistical heterogeneity between the studies was assessed using the Cochrane Q test (significance set at P<0.05) together with I^2^ values (significance set at I^2^>25%). Publication bias was represented as funnel plot and further assessed using the Egger’s test [26]. Sensitivity studies were performed by excluding individual studies repeatedly to identify any study significantly affected the overall results. All the statistical analyses were performed with Review Manager software version 5.1 and the Meta package for R (www.r-project.com).

## Results

### The Chinese Population Based Case-control Study

The baseline characteristics of the participants are shown in Table 1. The mean age of the controls was 55.3 years old and slightly older than the mean age of the cases (P = 0.001), and we found more normal women were at post-menopausal status (P<0.001). We also found that women in the case group were with earlier menarche age (P<0.001). Women with the oral contraceptives may affect the breast cancer risk (P = 0.045).

**Table 1:** The baseline characters for the participants in the study.

Characteristics	Controls (N = 580)	Cases (N = 593)	P-value
Age (year, ±SD)	55.3±9.3	53.0±11.3	0.001
Age at menarche (year, ±SD)	15.6±1.8	15.1±1.7	<0.001
Age at first birth (year, ±SD)	26.3±4.3	26.2±3.6	0.425
Menopausal status			<0.001
pre−/peri-menopausal	150 (26.3%)	216 (38.4%)	
post-menopausal	421 (73.7%)	347 (61.6%)	
Contraceptive users			0.045
yes	32 (5.5%)	49 (8.7%)	
no	548 (94.5%)	512 (91.3%)	
First-degree relatives’ history of breast cancer			0.693
yes	24 (4.1%)	27 (4.8%)	
no	556 (95.9%)	536 (95.2%)	
Season of blood collection			0.007
spring	204 (35.2%)	198 (33.4%)	
summer	220 (37.9%)	183 (30.9%)	
autumn	56 (9.7%)	81 (13.7%)	
winter	100 (17.2%)	131 (22.1%)	

In total, the mean 25(OH)D level for the normal women was 15.67 ng/ml and 11.31 ng/ml for the breast cancer patients (Wilcoxon’s test, P<0.001). Among the normal women, 80.0% showed vitamin D severe deficiency (less than 20 ng/ml), 15.2% have vitamin D deficiency status (20–30 ng/ml) and only 4.8% have sufficiency vitamin D in their blood (>30 ng/ml). However, the situation of vitamin D status for the breast cancer patients is worse with 96.1% of severe vitamin D deficiency, 3.2% of vitamin D deficiency and only 0.7% have sufficiency vitamin D in their blood sample (P<0.001). The mean 25(OH)D level was different among the seasons, with the highest 25(OH)D level was found in summer (17.76 ng/ml) and lowest in winter (10.75 ng/ml) for the normal women. The mean circulating 25(OH)D level was 15.67 ng/ml and 16.25 ng/ml in spring and autumn respectively for the normal women. For the breast cancer patients, the mean 25(OH)D was highest level in autumn (14.38 ng/ml) and lowest in the spring (10.16 ng/ml) with the mean 25(OH)D level was 11.24 ng/ml and 11.27 ng/ml in summer and winter. The seasonal 25(OH)D level was significantly lower for the breast cancer patients compared to normal women except the winter (Wilcoxon’s test, P<0.001 for spring, summer, autumn and P = 0.968 for winter).

We found a significantly inverse association between plasma concentration of 25(OH)D and breast cancer risk. Compared with the lowest quartile (≤10.4 ng/ml), women with higher 25(OH)D level showed a significantly reduced breast cancer risk with the adjusted OR was 0.43 (95% CI = 0.31–0.60) for Q2 (10.4–13.4 ng/ml), 0.24 (95% CI = 0.17–0.35) for Q3 (13.4–17.9 ng/ml) and 0.10 (95% CI = 0.06–0.15) for Q4 (≥17.9 ng/ml) and the p-trend value was less than 0.001 (Table 2). Additionally, when treated the 25(OH)D as a continuous variable in the multivariate model, we have found a significantly reduced risk for breast cancer with the OR was 0.84 (95% CI = 0.81–0.87, P<0.001) for per 1 ng/ml (equal to 2.5 nM) increment of plasma 25(OH)D level. In the stratification studies, we found that plasma 25(OH)D was significantly associated with a reduced breast cancer risk within each stratum including age (≥50 or <50 years), age at menarche (≥16 or <16 years), age at the first birth (≥26 or <26 years) and menopausal status (Table 3). In the stratified studies by the season of blood collect, we found a significant association of the blood 25(OH)D and breast cancer risk in spring, summer and autumn, whereas no such association was found in winter, which may be due to the relatively lower blood 25(OH)D level both for the breast cancer patients and the controls (Table 3).

**Table 2:** The odds ratios for breast cancer risk by plasma 25(OH)D concentration.

Plasma 25(OH)D	Cases		Controls		Crude model OR (95% CI)	Adjusted model* OR (95% CI)
Categorized (ng/ml)	No.	%	No.	%		
Q1 (≤10.4)	325	54.8	145	25.0	1.00 (Reference)	1.00 (Reference)
Q2 (10.4–13.4)	148	25.0	145	25.0	0.44 (0.32–0.60)	0.43 (0.31–0.60)
Q3 (13.4–17.9)	81	13.6	145	25.0	0.28 (0.19–0.39)	0.24 (0.17–0.35)
Q4 (>17.9)	39	6.6	145	25.0	0.11 (0.07–0.17)	0.10 (0.06–0.15)
p-trend					<0.001	<0.001
Continuous						
per 1 ng/ml increment	593		580		0.85 (0.82–0.88)	0.84 (0.81–0.87)

* The OR was adjusted by age, age at first birth, age at menarche, use of contraceptive, menopausal status, first-degree relatives’ history of breast cancer and season of blood collection.

**Table 3:** Stratification studies of the association between an increment of 1 ng/ml blood 25(OH)D and breast cancer risk.

Stratification	OR (95% CI)*	P value
Age		
<50	0.84 (0.79–0.90)	<0.001
≥50	0.85 (0.82–0.89)	<0.001
Menopausal status		
pre−/peri-menopausal	0.83 (0.77–0.89)	<0.001
post-menopausal	0.85 (0.82–0.89)	<0.001
Age at menarche		
<16	0.84 (0.80–0.87)	<0.001
≥16	0.85 (0.80–0.90)	<0.001
Age at first birth		
<26	0.86 (0.81–0.90)	<0.001
≥26	0.83 (0.79–0.87)	<0.001
Season of the blood collect		
Spring	0.79 (0.74–0.85)	<0.001
Summer	0.79 (0.74–0.84)	<0.001
Autumn	0.90 (0.81–1.00)	0.036
Winter	1.06 (0.97–1.17)	0.182

* Adjusted by age, age at menarche, menopausal status, age at first birth, season of the blood collect and the first-degree relatives’ history of breast cancer (excluding the stratified factors in each stratum).

### Meta-analysis of Blood 25(OH)D Level and Breast Cancer Risk

**Figure 1:**
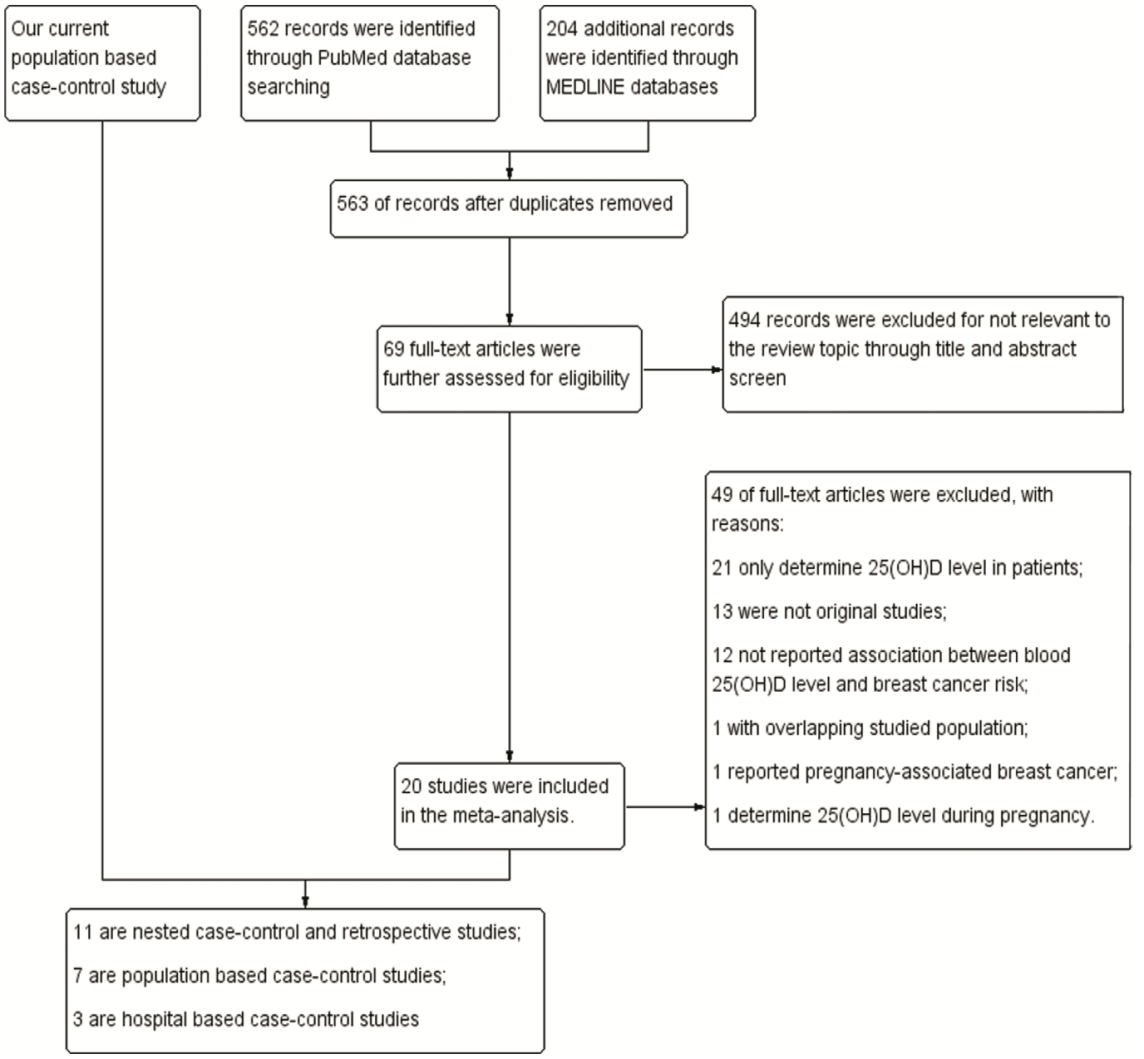
Working flow chart for selection of studies included in meta-analysis.

**Figure 2:**
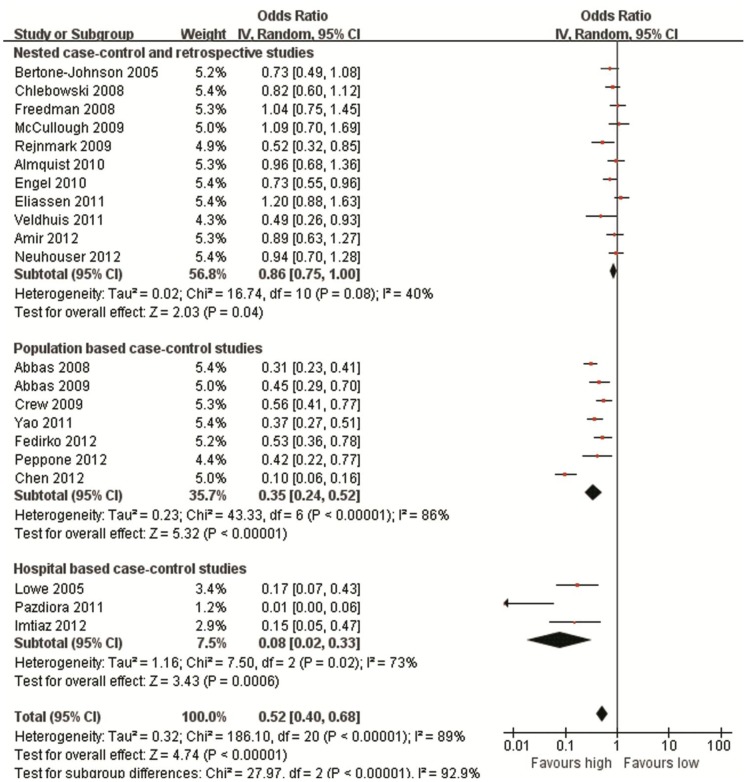
Forest plot of the highest quantile versus lowest quantile blood 25(OH)D level and breast cancer risk.

The working flow chart for the studies identification for the meta-analysis studies is shown as Figure 1. From the literature search, we have identified 21 eligibility studies that have evaluated the association between blood 25(OH)D level and the breast cancer risk (Table 4). 10 nested case-control [27], [28], [29], [30], [31], [32], [33], [34], [35], [36] and 1 retrospective studies [37] were found with a total of 6,811 cases and 9,041 controls, and three of these studies have reported a significantly inverse association for blood 25(OH)D level and breast cancer risk [31], [33], [37]. All the 7 included population based case-control studies (including 4,648 cases and 5,022 controls) [38], [39], [40], [41], [42], [43] and 3 hospital based case-control studies (including 312 cases and 483 controls) [44], [45], [46] reported an inverse relationship between blood 25(OH)D level and breast cancer risk. The risk estimates of breast cancer for the highest versus the lowest category of blood 25(OH)D level for individual studies and the summary estimates of the meta-analysis studies were shown in Figure 2. For the nested case-control and retrospective studies, the summary estimate for the highest quantile of blood 25(OH)D level versus the lowest quantile level indicated a significant reduced breast cancer risk (pooled OR = 0.86, 95% CI = 0.75–1.00) with moderate heterogeneity between the studies were identified (P = 0.08; I^2^ = 40%). The summary estimates for the population based case-control studies (pooled OR = 0.35, 95% CI = 0.24–0.52) and hospital based case-control studies (pooled OR = 0.08, 95% CI = 0.02–0.33) also suggested that women with higher blood 25(OH)D level showed a reduced breast cancer risk. Statistically significant heterogeneity among the subgroup studies was found (I^2^ = 92.9%, P<0.001; Figure 2). No significant publication bias was found for all meta-analysis studies as suggested by the Egger’s test (P = 0.133, prospective studies; P = 0.701, population based case-control studies; P = 0.121, hospital based case-control studies). Sensitivity studies indicated no single study significantly affects the results of the meta-analysis studies.

**Table 4:** Characteristics of the studies included in the meta-analysis of blood 25(OH)D and breast cancer risk.

Study(reference)	Country	Study type	No. of casepatients		No. of control subjects	Age, y	Measure of exposure	Adjusted OR(95% CI)#	Adjustments
Bertone-Johnson, 2005 (27)	United States	Nested	701		724	30–55	Plasma: ≥48 vs. ≤28 ng/mL	0.73 (0.49–1.07)	Age, menopausal status, postmenopausal hormones use, blood collection time, fasting status, BMI, parity/age at first birth, family history, benign breast disease, age at menarche, age at menopause, alcohol, plasma a-carotene
Chlebowski, 2007 (28)	United States	Nested	1067, postmenopausal		1067	50–79	Serum: <32.4 vs. ≥67.6 nM	1.22 (0.89–1.67)	Age, race/ethnicity, latitude, venipuncture, HRT use, dietary, BMI, physical activity, family history, breast biopsy, estrogen plus progestin use, estrogen use
Freedman, 2008(29)	United States	Nested	1005, postmenopausal		1005	55–74	Serum: ≥33.7 vs. <18.3 ng/mL.	1.04 (0.75–1.45)	Blood draw period, age, serum collection season, BMI, age at menarche, age at menopause, HRT use, benign breast disease, family history, parity and age at first birth, smoking, alcohol, calcium
McCullough, 2009 (30)	United States	Nested	516		516	47–85	Serum: ≥73.2 vs. <36.7 nM	1.09 (0.70–1.68)	Birth year, blood draw, race, season, parity and age at first birth, BMI, weight change.
Rejnmark, 2009(31)	Denmark.	Nested	142		420	Mean age: 58 (cases and controls)	Plasma: >84 vs. <60 nM	0.52 (0.32–0.85)	Not reported.
Almquist, 2010(32)	Sweden	Nested	764		764	Mean age: 57 (cases and controls)	Serum: ≥107 vs. <71 nM	0.96 (0.68–1.37)	BMI, education, socioeconomic index, alcohol, smoking, marital status, birth country, age at menarche, oral contraception use, children number, HRT use, PTH, calcium, albumin, creatinine and phosphate.
Engel, 2010 (33)	France	Nested	636	1272		Mean age: 56.9 (cases and controls)	Serum: >27 vs. <19.8 ng/ml	0.73 (0.55–0.96)	Age, menopausal status, age at menopause, study center, blood collection date, BMI, physical activity, age at menarche, number of children, tobacco, oral contraceptives use, menopausal hormone therapy use, mammography, benign breast disease, family history, alcohol, energy, calcium and vitamin D dietary and supplement intakes, serum calcium, PTH, estradiol, and progesterone concentrations
Eliassen, 2011 (34)	United States	Nested	613	1218		25–42	Plasma: ≥30.6 vs. <18.4 nM	1.20 (0.88 to 1.63)	Age at menarche, BMI, parity and age at first birth, family history, benign breast disease, age at blood collection, blood collection date and time, fasting status, luteal day, race, menopausal status
Veldhuis, 2011(37)	Netherlands	Retrospective	56	119		Not reported	Serum: ≥50 vs. <50 nM	0.49 (0.41–0.77)	None
Amir, 2012 (35)	United States and Canada	Nested	231	856		>35	Serum: <72 vs. ≥72 nM	1.25 (0.88–1.77)	None.
Neuhouser, 2012 (36)	United States	Nested	1080	1080		50–79	Serum: <36.7 vs. ≥64.9 nM	1.06 (0.78–1.43)	WHI intervention arm, BMI, physical activity, smoking, mammography within the past 2 years, Gail 5-year risk score, HRT use, alcohol intake
Abbas, 2008 (38)	Germany	Population based	1394, postmenopausal	1365		50–74	Serum: ≥75 vs. <30 nM	0.31 (0.24–0.42)	Blood collection, year of birth, age at menopause, family history, benign breast disease, pregnancies number, age at menarche, breastfeeding, mammograms number, HRT use, BMI, education, smoking
Abbas, 2009 (39)	Germany	Population based	289, premenopausal	595		30–50	Plasma: ≥60 vs. <30 nM	0.45 (0.29–0.70)	Age, blood collection time, births number, family history, age at menarche, breast-feeding, BMI, alcohol
Crew, 2009 (40)	United States	Population based	1026	1075		Mean age: 58.6 (cases), 56.1 (controls)	Plasma: >40 vs. <20 ng/ml	0.56 (0.41–0.78)	Age, race, age of menarche, age of first birth, parity, breastfeeding history, menopausal status, HRT use, family history, benign breast disease, BMI, physical activity, blood draw season
Yao, 2011 (41)	United States	Population based	579	574		Not reported	Serum: ≥30 vs. <20 ng/ml	0.37 (0.27–0.51)	Age, BMI
Fedirko, 2012 (42)	Mexico	Populationbased	573	639		35–69	Serum: >25 vs. ≤20 ng/ml	0.53 (0.36–0.78)	Age, health care system, region, blood draw season, socioeconomic status, alcohol, family history, parity/number of children born alive, age at first full term pregnancy, breast-feeding, HRT use, BMI, height, physical activity, energy, menopausal status
Peppone, 2012 (43)	United States	Populationbased	194	194		40–70	Serum: <20 vs. ≥32 ng/ml	2.41 (1.30–4.48)	Age, laboratory, blood draw month
Lowe, 2005 (44)	United Kingdom	Hospital based	179	179		34–84	Plasma: <50 vs. >150 nM	5.83 (2.31–14.7)	None
Pazdiora, 2011 (45)	Czech Republic	Hospital based	43	214		Mean age: 61 (cases), 65 (controls)	Serum: >25 vs. <15 ng/mL	0.01 (0.001–0.058)	None
Lmtiaz, 2012 (46)	Pakistan	Hospital based	90	90		Mean age: 47.5 (cases), 46.2 (controls)	Serum: ≥20 vs.<20 ng/ml	0.15 (0.05–0.47)	None

* Abbreviations: OR, odds ratio; 95% CI, 95% confidence interval; BMI, body mass index; HRT, hormone replacement therapy; PTH, parathyroid hormone; WHI, women’s health initiative.

# Odds ratio for the highest versus lowest category of blood 25(OH)D level.

## Discussion

From our population study, we found that vitamin D deficiency was a common phenomenon for the women and lower blood vitamin D level may increase the breast cancer risk in the Chinese population. Vitamin D deficiency and insufficiency could be found in nearly 95% of the general women with the plasma 25(OH)D concentration was less than 30 ng/ml. A dual-centered study conducted by Woo et al. in China found that more than 90% of young women in Beijing and Hong Kong had circulating 25(OH)D level less than 50 nmol/L (equal to 20 ng/ml) [47]. Lu et al. found that 69.2% of middle-aged and elderly Chinese individuals (including women and men) with the plasma 25(OH)D level less than 50 nmol/L [48]. Unlike the western populations, women in China rarely take vitamin supplements and there is also a lack of vitamin D fortified food, which lead to the relatively lower plasma 25(OH)D level compared to the western women. We found that the mean plasma vitamin D level was usually higher when collected in summer and relatively lower in winter, for which could be due to that a majority of 25(OH)D were synthesized by the skin under sun exposure. Significantly lower plasma 25(OH)D level was found for the breast cancer patients compared to the normal women population when the blood was collected in spring, summer or autumn; however, no significant difference was found for the mean plasma 25(OH)D level between the two groups when the blood was collected in winter, which may also be due to the relatively lower 25(OH)D level for the participants.

Experimental studies have revealed that 1α,25-(OH)_2_D could inhibit cell proliferation, promote cell differentiation, inhibit the tumor cell metastasis and angiogenesis, suggests an anticancer activity for the vitamin D metabolite [13]. As the major storage form for the vitamin D in the body, 25(OH)D are considered a valuable marker for vitamin D status because it incorporates all sources of vitamin D intake including those parts that are synthesized by the skin. 25(OH)D is the substrate for the synthesis of 1α,25-(OH)_2_D in the kidney nephrons and possibly also in breast tissue. Many epidemiological studies have been conducted to evaluate the association for the blood 25(OH)D and/or 1α,25-(OH)_2_D level and the risk for breast cancer. Our previous systematic review suggested that no significant association between blood 1α,25-(OH)_2_D level and breast cancer risk [23], which may be due to that 1a,25(OH)_2_D is under tight regulation by 1a-hydroxylase which is under the control of the parathyroid hormone (PTH) in response to serum calcium. For blood 25(OH)D level, 7 population based case-control studies and 3 hospital based case-control studies consistently reported an inverse relationship between blood 25(OH)D level and breast cancer risk; however, the blood sample for the breast cancer patients was collected after the diagnosis. It not clear that whether cancer diagnosis may have influenced circulating 25(OH)D through treatment or behavioral changes; however, the blood samples for the patients were usually collected shortly after the diagnosis and prior to any treatment. We cannot exclude the possibility that the presenting of breast cancer cells may influence the blood 25(OH)D level. From the systematic literature search, we have identified 10 prospective nested case-control studies have evaluated the pre-diagnostic blood 25(OH)D level and breast cancer risk. Rejnmark et al. observed a 48% (OR = 0.52, 95% CI = 0.32–0.85) reduced breast cancer risk for those with a plasma 25(OH)D level over 84 nM compared to those less than 60 nM for the Danish women; however, significant reduced breast cancer was only found for the premenopausal women (OR = 0.38, 95% CI = 0.15–0.97) but not for the postmenopausal women (OR = 0.71, 95% CI = 0.38–1.30) in the stratification study [31]. With a nested case-control study within the French E3N cohort, Engel et al. found that increasing 25(OH) vitamin D3 serum concentrations lead to a reduced breast cancer risk (P-trend = 0.02) with the odds ratio was 0.73 (95% CI = 0.55–0.96) for women in the highest tertile compared to the lowest tertile [33]. In a nested case-control analysis of 701 cases and 724 controls from the Nurses’ Health Study cohort, Bertone-Johnson et al. found that women in the highest quantile of 25(OH)D had a marginal 27% reduced breast cancer risk compared with those of the lowest quantile (OR = 0.73, 95% CI = 0.49–1.07; P-trend = 0.06) [27]. The rest of the other nested case-control studies reported no significant association between the blood 25(OH)D level and breast cancer risk. Another retrospective study conducted by Veldhuis et al. found that more breast cancer patients have a plasma 25(OH)D level less than 50 nM compared to the women with normal bone density indicates a protective effects against breast cancer risk for vitamin D [37]. From the meta-analysis, we found that a higher pre-diagnostic blood 25(OH)D level showed a weaker but significant protective effect for the breast cancer risk compared to the results from the hospital or population based case-control studies. These data strongly suggested that high blood vitamin D level may have protective effects against breast cancer.

Vitamin D deficiency is common for breast cancer patients and it may be correlated with poorer prognosis. Breast cancer patients with early-stage have higher vitamin D level than those of advanced stage disease [49]. For the premenopausal breast cancer patients, the 25(OH)D was significantly lower for those with high-grade tumors, ER negative tumors and the 25(OH)D level was lowest for those of triple negative tumors [41]. Goodwin et al. firstly reported that early-stage breast patients with vitamin D deficiency (<50 nmol/L) showed an increased risk of distant recurrence (hazard ratio [HR] = 1.94, 95% CI = 1.16–3.25) and death (HR = 1.73, 95% CI = 1.05–2.86) compared with those with sufficient vitamin D levels (>72 nmol/L) [18]. Kim et al. found that vitamin D deficiency (<20 ng/ml) was associated with poorer outcomes for patients of luminal-type [50]. Vrieling et al. reported that lower serum 25(OH)D concentrations was associated with poorer overall survival and distant disease-free survival in postmenopausal breast cancer patients [51]. However, there is still a lack of evidence to support the use of vitamin D supplementation as adjuvant therapy for cancer treatment will benefit the patients. A pioneer study conducted by Crew et al. found that even premenopausal breast cancer patients (>75% with plasma 25(OH)D level less than 50 nmol/L) were supplied with 400 IU vitamin D3 daily for 1 year, only a small fraction of the patients (<15%) would achieved sufficiency vitamin D level (>75 nmol/L) [52]. Another study conducted by Vashi et al. found that when provided an oral 8000 IU/day of vitamin D3 for 8 weeks to those breast cancer patients with serum 25(OH)D level less than 32 ng/ml, only 46.7% showed an increased blood 25(OH)D level over 32 ng/ml [53]. The low response rate to the vitamin D supplementation for the breast cancer patients may be due to the chemotherapy received by the patients that may reduced the absorption of vitamin D or may be due to that the baseline serum 25(OH)D level was relatively lower for the patients and more vitamin D supplementation are warranted.

We acknowledged there are several limitations for the current study. Firstly, the design of our population based case-control study is more prone to a variety of biases such as selection bias and recall bias, and the results could not excluded the possibility that lower vitamin D level for the patients was affected by the presenting of cancer cells. Although the data from the nested case-control studies suggested an anticancer activity for the higher blood 25(OH)D level, we also could not directly come to the conclusion that high vitamin D supplementation would have reduced the breast cancer risk. It should be confirmed by other well designed observational studies such as cohort studies as well as by randomized clinical trials. A reduced of overall cancer risk was found when vitamin D (approximately 1,100 IU/d) and calcium (1,400 mg/d) were simultaneously administered to postmenopausal women in a clinical trial conducted by Lappe et al., although only a few breast cancers occurred in either subgroup [54]. In another random clinical trial, vitamin D supplementation (400 U/d) was not associated with reduced breast cancer risk from the Women’s Health Initiative study, which may be due to the relatively low dose of vitamin D supported to the participants [28]. Thus, more randomized clinical trials with sufficient statistical power are warranted to address the questions. Secondly, although the meta-analysis has included a total of 11 nest case-control and retrospective studies, 7 population based case-control studies and 3 hospital based case-control studies. A relatively smaller total number of the participants were recruited. Significantly heterogeneity between the study design for the pooled estimates was found (P<0.001). More observational studies are warranted to get a more precise anticancer activity for the breast cancer risk. Thirdly, vitamin D has been found to be associated with other factors such as outdoor physical activity and fitness, which may be partially attribute to the association between vitamin D and breast cancer risk.

In conclusion, our study suggested that higher blood vitamin D level was associated with a significantly reduced risk of breast cancer. Considering the vitamin D deficiency is common in populations, women will likely to be benefited by taking sufficient vitamin D with the goal of reducing the breast cancer risk; however, more studies should be conducted before we could achieve the goals.
